# Jawless land snail *Sinorachis*, a new bradybaenine genus from China (Eupulmonata, Camaenidae)

**DOI:** 10.3897/zookeys.893.38445

**Published:** 2019-12-02

**Authors:** Min Wu, Zheyu Chen, Liwan Zhang

**Affiliations:** 1 School of Life Sciences, Nanjing University, Xianlindadao 163, Qixia, Nanjing 210023, China Nanjing University Nanjing China; 2 College of Food Science and Engineering, Wuhan Polytechnic University, Wuhan 430023, China Wuhan Polytechnic University Wuhan China; 3 Design Team of Forest Inventory and Planning of Lichuan, Lichuan 445400, Hubei, China Design Team of Forest Inventory and Planning of Lichuan Lichuan China

**Keywords:** Bradybaeninae, Enoidea, Hubei, taxonomy

## Abstract

A new land snail species that represents a new genus is reported from Hubei, China. The snail has a conical shell with pits and/or granules on embryonic whorls and a smooth teleoconch with straight peristome. The head of the animal has a developed wart. The mantle lobe is only developed on the left side. At the front of the buccal mass there is no chitinous jaw that is without exception seen in Chinese camaenids. Its radular teeth are usually slender and tongue-shaped, not typical in bradybaenine snails. The genital system is typical of Bradybaeninae and is characterized by the absence of a membranous sac surrounding the terminal genitalia, penial caecum or flagellum; a well-developed penis sheath; a symmetrical dart sac apparatus; and one distally branched mucous gland. The new species *Sinorachis baihu* Wu & Chen, **gen. and sp. nov.**, is assigned to the type species of the new genus, in which all the known Chinese *Rachis* species are included. Thereby, the new genus is composed of three species, namely *Sinorachis onychinus* (Heude), **comb. nov.**, *Sinorachis aureus* (Heude), **comb. nov.** and the new species.

## Abstract (cn)

从湖北报道了一个陆生贝类新物种并指定其为新属华霜螺属*Sinorachis* Wu & Chen, **gen. nov.** 的模式种。该属因生殖系统具有巴蜗牛亚科（坚螺科）的典型矢囊结构而被列入巴蜗牛亚科。模式种白虎华霜螺*Sinorachis baihu* Wu & Chen, **gen. and sp. nov.** 口球前端的颚片阙如，系已知中国的坚螺科中首个口球缺乏颚片的物种。此外，白虎华霜螺齿舌的齿多呈细舌状且排列紧密，与常见的巴蜗牛亚科的齿舌不同。华霜螺属的鉴别特征为：贝壳锥形；胚螺层具微小凹坑和/或颗粒；成螺光滑；壳口缘不反折。头瘤发达；外套膜左缘具小叶。颚片阙如。生殖系统基部膜囊缺乏；具交接器鞘；鞭状体无；矢囊器官对称；粘液腺一根、分支。几何形态学分析亦表明本属具有与霜纳螺*Rachis* Albers、中国艾纳螺各属及巴蜗牛亚科的拟锥螺*Pseudobuliminus* Gredler相异的贝壳特征。由之将中国原艾纳螺总科霜纳螺属的所有物种移入本新属。华霜螺属由爪华霜螺*Sinorachis onychinus* (Heude), **comb. nov.**、金华霜螺*Sinorachis aureus* (Heude), **comb. nov.** 与本新种组成。

## Introduction

*Rachis* Albers, 1850 (type species *Bulimus punctatus* Anton, 1838; SD Martens in Albers 1860) is an enoid genus comprised of many known species from Africa and India (Schileyko 1998b). Two Chinese land snail species with conical shells were assigned to this genus based on shell morphology (Gredler 1887; Yen 1939; Wu 2018). Although Möllendorff (1901) placed them in *Buliminus* and thought they appeared to be related to the group, including *Bulimus cantori* Philippi, 1844 (= *Mirus cantori*), he wondered about the absence of an angular nodule that is ubiquitously observed in the Chinese enids. The Chinese *Rachis* species differed from the true *Rachis* species by the presence of embryonic shell sculpture that is unseen in *Rachis* (Schileyko 1998b). However, this sculpture may have been overlooked by early researchers.

The shells of Chinese *Rachis* species are, in many respects, untypical of the bradybaenine genera. In our recent work on the land snails from Hubei Province, comparison of the shell morphology revealed an arboreal snail that was firmly believed to fall into the same category of Chinese *Rachis*. The genital pattern of this species, however, totally differs from those of these enoids; rather, the developed dart sac apparatus suggests it belongs to the camaenid subfamily Bradybaeninae.

## Materials and methods

Living specimens were relaxed by drowning in fresh water before being transferred to 70% ethanol for fixation, which was replaced with ethanol of the same concentration after three days. Just after the specimens were relaxed, a piece of foot was cut and preserved in 99.7% ethanol for future molecular analysis. The shell and genitalia were measured with digital vernier calipers and from photographs to the nearest 0.1 mm. Whorl number was recorded as described by Kerney and Cameron (1979), with 0.125 (= ^1^/_8_) whorl accuracy. Soft parts were measured after the specimens were sufficiently fixed in 70% ethanol. Directions used in descriptions: proximal = towards the genital atrium; distal = away from the genital atrium.

The buccal mass was removed and treated in 10% sodium hydroxide solution under 60 degrees Celsius for up to 10 min before extracting the radula, which was cleaned by water using an ultrasonic cleaner. Then the radula was transferred into 75% ethanol. Radulae and shell were examined under a scanning election microscope (Sigma 500).

Geometric morphometric methods were used to explore the conchological relationship among the new species described herein, the enoids and the high-shelled bradybaenine species distributed in mainland China. Shell morphological variation analyses were performed in the tps series software including tpsUtil32 (Rohlf 2004) and tpsDig32 (Rohlf 2005), using the geometric morphometric methods based on the landmarks and semi-landmarks on the profile of the aperture-viewed shell as per Schilthuizen et al. (2012). The designs of the landmarks and semi-landmarks are as follows (Fig. 9B):

**LM1** the crossing of peristome and left profile of body whorl;

**LM2** the columella insertion;

**LM3** the right insertion of peristome onto body whorl;

**LM4** and **LM10** the right and left terminal points on last suture, respectively;

**LM5** and **LM9** the right and left terminal points on penultimate suture, respectively;

**LM6** and **LM8** the right and left terminal points on suture above the penultimate one, respectively;

**LM7** apex of shell;

**LM11–18** eight semi-landmarks on the left profile between LM10 and LM1 by length;

**LM19–36** eighteen semi-landmarks on the peristome between LM1 and LM3 by length.

The usually used landmark point crossed by the right profile and the last part of suture (arrowed on Fig. 9B) was not chosen in this study because the point is not present on all the aperture-view images of the specimens observed herein. The landmarks and the semi-landmarks were treated indiscriminately. The geometric morphometric analysis employed aperture-viewed images of a total of 232 shells including most of Chinese enids (112 specimens of 112 species, including one from Wu and Xu 2011, 111 from Wu 2018; see Appendix 1), some Chinese *Pseudobuliminus* Gredler, 1886 and one *Stenogyropsis* Möllendorff, 1899 species (102 specimens of 20 species: SMF and HBUMM specimens; see Appendix 1), *Rachis* Albers, 1850 (five specimens of two species: SMF specimens and Raheem et al. 2014: fig. 39a; see Appendix 1) and *Rhachistia* Connolly, 1925 (seven specimens of four species in Raheem et al. 2014: figs 39B–40B), and *Sinorachis* gen. nov. (five specimens of three species: SMF and HBUMM specimens; one image of *Buliminus aureus* Heude, 1890 from Heude 1890: Pl. 35, fig. 21). The detailed information of the specimens used in this work is listed in the Appendix 1. Full Procrustes fitting, covariance matrix generation, and subsequent canonical variate analysis (CVA) were performed using MorphoJ (version 1.05f; Klingenberg 2011).

Abbreviations used in the text and figures are as follows:

**A** anus;

**AS** accessory sac, a sac both inserted by mucous glands and opening into the chamber containing the love dart or opening into the dart sac chamber;

**At** atrium;

**AU** auricle;

**BC** bursa copulatrix;

**BCD** bursa copulatrix duct;

**DS** dart sac;

**DtC** love dart chamber, the chamber secreting and containing the love dart;

**Ep** epiphallus;

**FO** free oviduct;

**HBUMM** mollusk collection of the Museum of Hebei University, Baoding, China;

**HG** hindgut;

**K** kidney;

**MC** mantle collar;

**MG** mucous glands;

**P** penis;

**PC** pericardium;

**PG** pallial gland;

**PR** penial retractor muscle;

**PS** penis sheath;

**PV** principal pulmonary vein;

**SMF** Forschungsinstitut und Naturmuseum Senckenberg, Frankfurt;

**U** ureter;

**UO** ureteric orifice;

**V** ventricle;

**Va** vagina;

**VD** vas deferens.

## Systematics

### Helicoidea Rafinesque, 1815

#### Camaenidae Pilsbry, 1895

**Bradybaeninae Pilsbry, 1898**

##### Sinorachis

Taxon classificationAnimaliaAsparagalesOrchidaceae

Wu & Chen gen. nov.

D4AC8A9B-9F39-549C-BB6B-91FF668E4CC6

http://zoobank.org/98AD47D3-B40F-4EE7-9678-5F9657D0FCD4

###### Type species.

*Sinorachis baihu* Wu & Chen, gen. and sp. nov.

###### Diagnosis.

Shell conical. Embryonic shell with pits and/or granules. Adult shell smooth. Peristome not reflexed. Head wart developed. Mantle lobe only present on left side. Jaw absent. Membranous sac surrounding terminal genitalia absent. Penis sheath present. Penial caecum absent. Flagellum absent. Dart sac apparatus symmetrical. Mucous glands one; branched.

###### Description.

***Shell*** conical. Whorls slightly convex. Suture impressed. Protoconch brownish purple; shiny; with tiny pits and/or granules. Adult shell smooth, not hairy or scaly. Body whorl large. Peristome not reflexed. Aperture not expanded. Umbilicus a slit. Shell glossy; banded or not.

***General anatomy*.** Eversible head wart between ommatophore insertions developed. Lobe on mantle collar present on left but absent on right. Jaw absent. Crop thin, indistinguishable from the remaining alimentary tract.

***Pallial complex*.** Ureter closed. Kidney triangular, not bilobed.

***Genitalia*.** Penis sheath present. Penis externally simple; internally with several pilasters. Flagellum absent. Epiphallus and vas deferens distinctly demarcated. Membranous sac surrounding terminal genitalia absent. Dart sac apparatus symmetrical. Accessory sac present. Poly-layered structure developed in dart sac. Mucous gland branched; inserting into dart sac through one peduncle.

###### Etymology.

This new genus is named after *sino* (China) and *rachis*, an enoid genus in which the old species of the new genus were placed.

###### Distribution.

**Figure 1.**
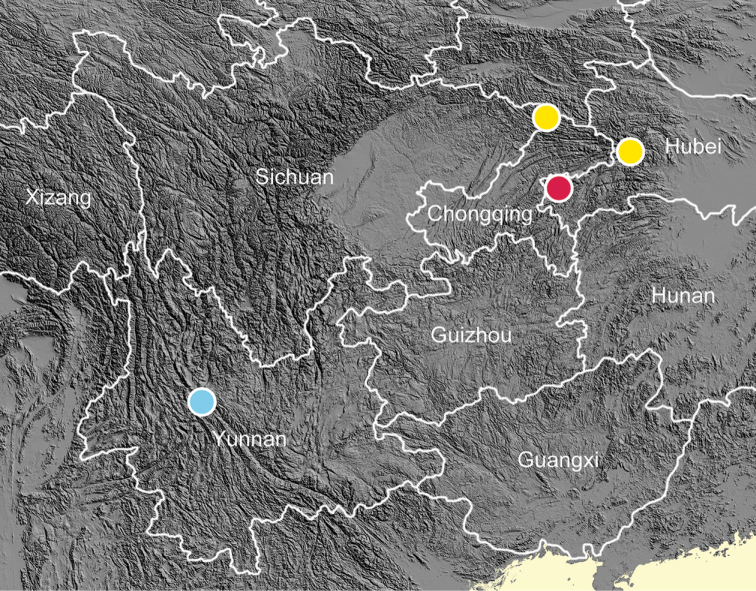
Distribution map of *Sinorachis* Wu & Chen, gen. nov. Blue dot, *Sinorachis aureus* (Heude, 1890) comb. nov.; yellow dots, *Sinorachis onychinus* (Heude, 1885) comb. nov.; red dot, *Sinorachis baihu* Wu & Chen, gen. and sp. nov.

Hubei (Badong, Lichuan), Chongqing (Chengkou), Yunnan (Dali) (Fig. 1).

###### Remarks.

*Sinorachis baihu* Wu & Chen, gen. and sp. nov. shares most conchological features with the other two known species, namely *Buliminus onychinus* Heude, 1885 and *Buliminus aureus* Heude, 1890, which were treated by some authors as species of the enoid genus *Rachis* (Gredler 1887). But based on pallial complex and genital system, the new species described herein should not been assigned to the genus *Rachis* Albers, 1850 (Enoidea) that belongs to Orthurethra and there is no dart apparatus in genitalia. The new genus is entirely in character for the subfamily Bradybaeninae in possessing the typical dart sac apparatus that does not differ from that commonly seen in all the Chinese bradybaenine genera. In our opinion, based on shell morphology, *B. onychinus* and *B. aureus* should also belong to the genus *Sinorachis* gen. nov.

The new genus is the only one that lacks a jaw in the subfamily Bradybaeninae. Like *Bradybaena* Beck, 1837 and some other bradybaenine genera (Wu et al. 2019), the genus shows a leaf-shaped appendage on the left mantle collar. The shells of the new genus are quite different from those of the high-shelled genus *Pseudobuliminus* Gredler, 1886 in having a sculptured embryonic shell and a distinctly large body whorl. In aspects of general shell morphology, the shell of the new genus, the genus *Rachis* Albers, 1850 distributed in Africa and India, the genus *Rhachistia* Connolly, 1925 distributed in eastern Africa and Asia, Chinese enid genus and Chinese *Pseudobuliminus* spp. can be discriminated with the aid of the geometric morphometric methods (Fig. 10) based on the landmarking scheme employed herein (Fig. 9B).

In comparison with Chinese species of another bradybaenine genus, *Pseudobuliminus*, that also has a high spired adult shell and embryonic sculpture, the new genus has a poly-layered structure, an accessory sac, and a single branch of mucous gland in the dart sac apparatus, but has no membranous sac surrounding terminal genitalia. If only focusing on the characteristic spectrum of genitalia (table 1, in Wu 2019), the genus is closest to *Ponsadenia* Schileyko, 1978 but these two genera can be distinguished by presence/absence of the poly-layered shell structure and the structure of accessory sac which looks like a bridge in the latter genus.

##### Sinorachis baihu

Taxon classificationAnimaliaAsparagalesOrchidaceae

Wu & Chen sp. nov.

257DD29C-258B-5E2B-89E1-76C71C8B3183

http://zoobank.org/9C040E3B-E506-4B4A-B3F7-7AD55704A91F

Figs 1 , 2 , 3 , 4 , 5 , 6 , 7 , 8 , 9 , 10

###### Type material.

***Holotype***: CHINA • fully mature animal; Hubei, Lichuan, Liangwuxiang, Shanchacun; 108.837°E, 30.274°N; 1.XI.2018; Liwan Zhang leg.; HBUMM08296-specimen 1. ***Paratype***: one juvenile animal; same data as for preceding; HBUMM08296-specimen 2. Partial foot was cut off in both specimens and preserved in 99.7% ethanol at -20 °C; HBUMM08296a-specimens 1, 2.

###### Diagnosis.

Embryonic shell with pits, each having a central hump. Shell with three bands.

###### Description.

**Figure 2.**
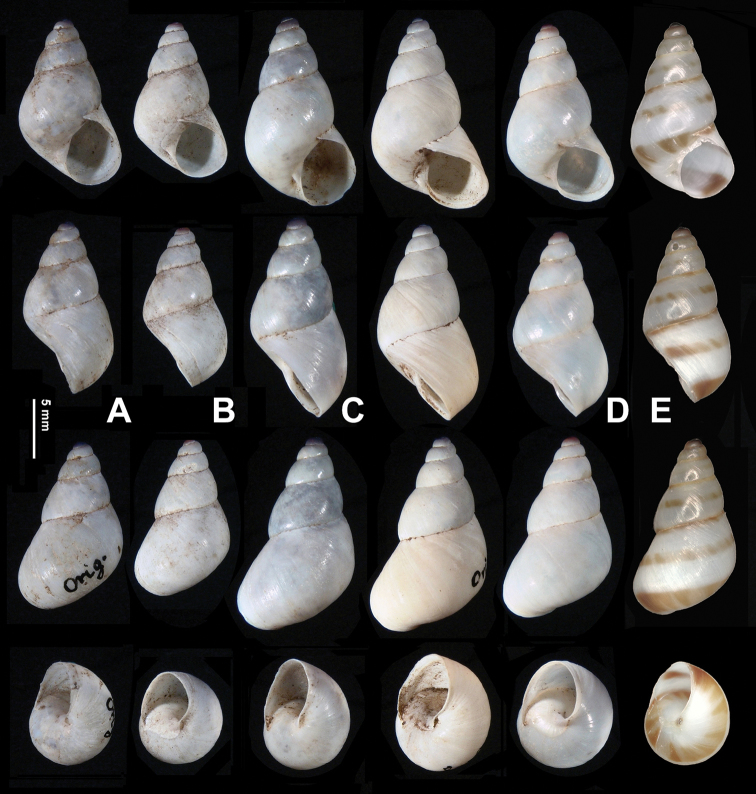
**A–D***Sinorachis onychinus* (Heude, 1885) comb. nov., shells **A** SMF42825 **B** SMF42826 **C** SMF42827, SW Hupei **D** SMF104593 **E***Sinorachis baihu* Wu & Chen, gen. and sp. nov., HBUMM08296-specimen 1, holotype.

***Shell*** (Figs 2, 6D, E). Conical; thin but solid; dextral. Whorls slightly convex. Suture impressed. Umbilicus a slit. Columella almost vertical. Protoconch densely and evenly covered with fine centrally-uplifted pits (Fig. 6D, E). Teleoconch without spiral furrows. Aperture oblique; not sinuate at peristome. Body whorl not descending behind aperture. Shell surface without ribs. Growth lines fine. Adult shell not hairy or scaly. Adult body whorl rounded at periphery; with bottom convex. Ring-like thickening within aperture absent. Peristome thin; not reflexed. Callus thin and transparent. Shell glossy; white. A suture band, a supra-peripheral band and a subperipheral band more or less broken. Measurements (holotype): shell height 14.7 mm, shell breadth 8.7 mm, aperture height 5.3 mm, aperture width 3.5 mm, embryonic shell whorls 1.500, whorls 5.125, shell height/ breadth ratio 1.70.

**Figure 3.**
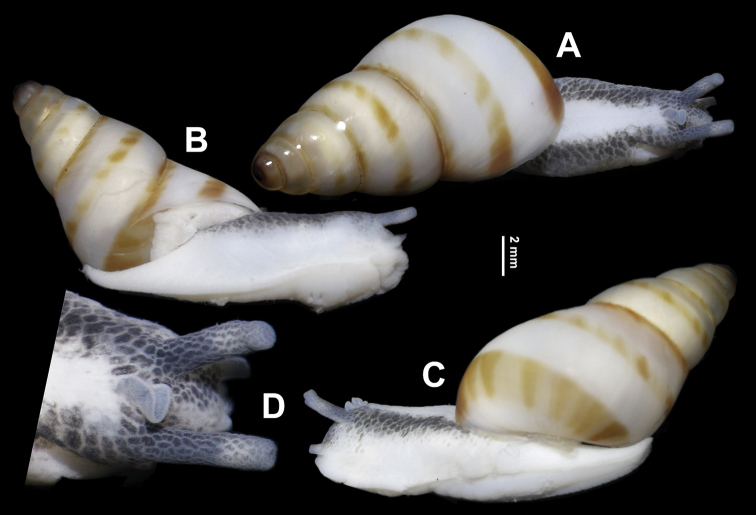
*Sinorachis baihu* Wu & Chen, gen. and sp. nov., HBUMM08296-specimen 1, holotype **A–C** living animal **D** magnified view of head.

**Figure 4.**
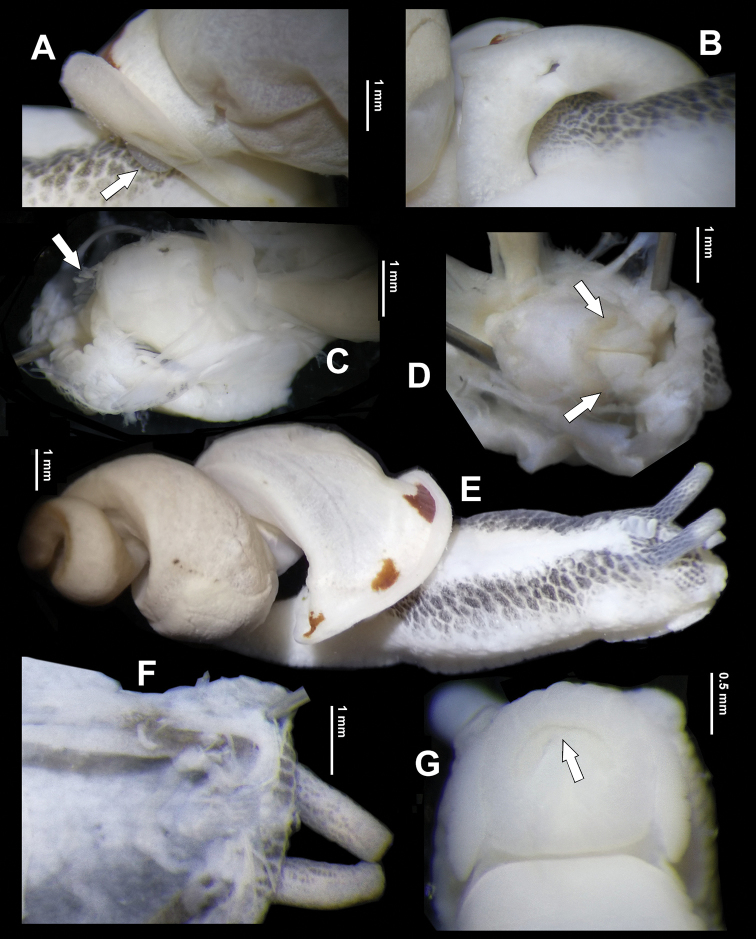
**A–F***Sinorachis baihu* Wu & Chen, gen. and sp. nov., HBUMM08296-specimen 1, holotype. **A** left margin of mantle collar with the lobe arrowed **B** right margin of mantle collar **C** lateral dorsal view of buccal mass with an oral curtain (a sheet of curtain-like tissue on the most anterior of the buccal mass) arrowed **D** dorsal view of buccal mass with an oral curtain arrowed **E** shell-removed animal, showing three pigmentation patches near mantle margin **F** internal body wall of head, showing no obvious head gland between the ommatophore tentacles **G** HBUMM08296-specimen 2, paratype, mouth of ventral view with an oral curtain arrowed.

***General anatomy*** (Figs 3, 4). A crest-like head wart between and slightly behind ommatophore insertions present. On left edge of mantle collar a leaf-shaped appendage present. Body dorsally white; symmetrically with two lateral black pigmented stripes that become lighter near sole. Sole creamy white. Jaw absent (Fig. 4C, D, G).

**Figure 5.**
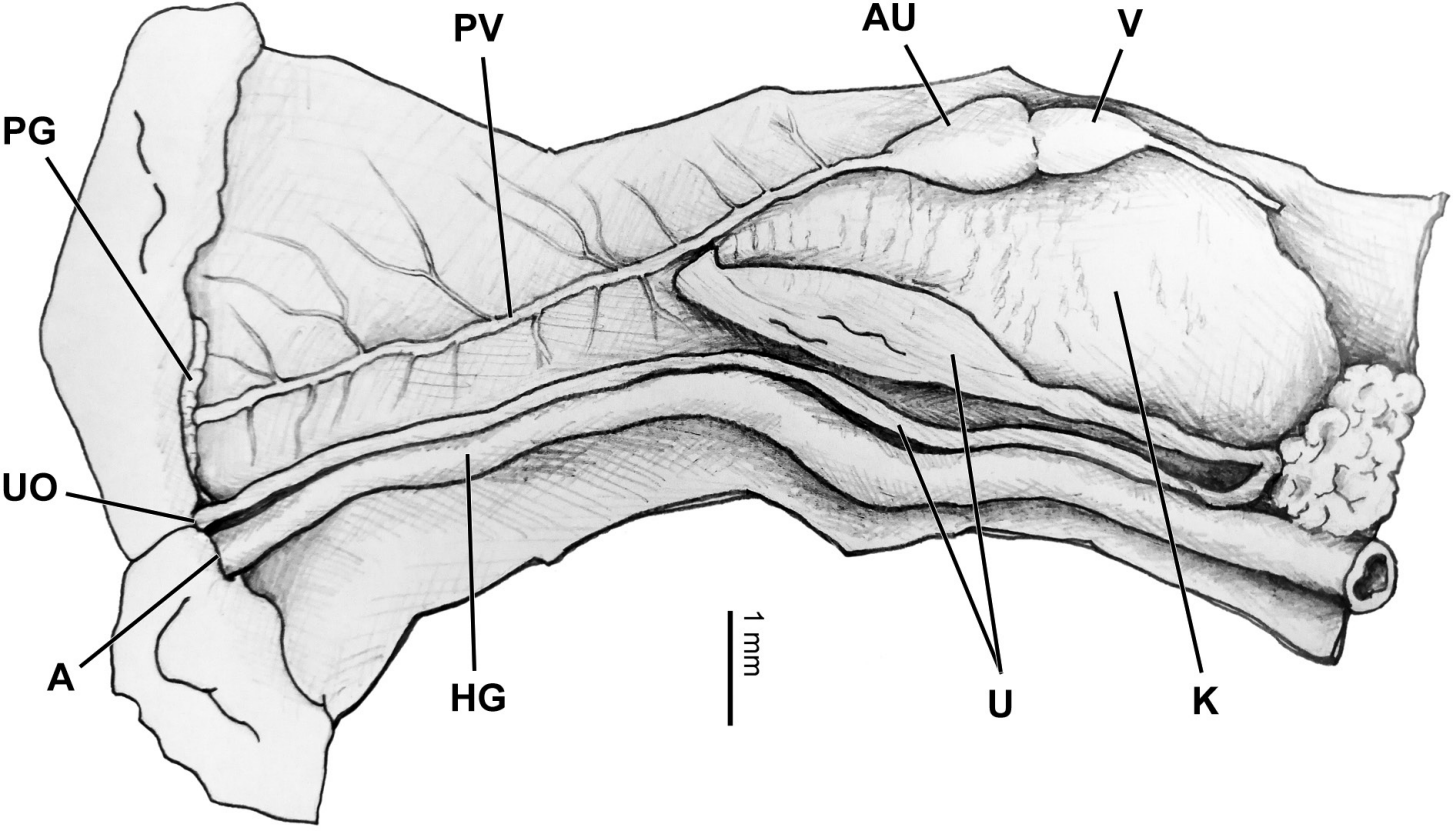
Pallial complex of *Sinorachis baihu* Wu & Chen, gen. and sp. nov., HBUMM08296-specimen 2, paratype.

***Pallial complex*** (Fig. 5). Pallial roof not pigmented. Pallial gland thin, parallel to mantle collar. Hindgut running parallel to parietal-palatal margin for length of pallial chamber. Ureter slender, typical sigmurethrous, about 1/5 breadth of hindgut, adhering to hindgut for all its length. Secondary ureter developed. Kidney triangular, not bilobed, about as long as 1/2 of pallial chamber. Heart as long as 1/3 – 1/2 of kidney. Main pulmonary vein running along contour and apex of kidney, then diffusing into thinner veins mostly concentrated on anterior half.

**Figure 6.**
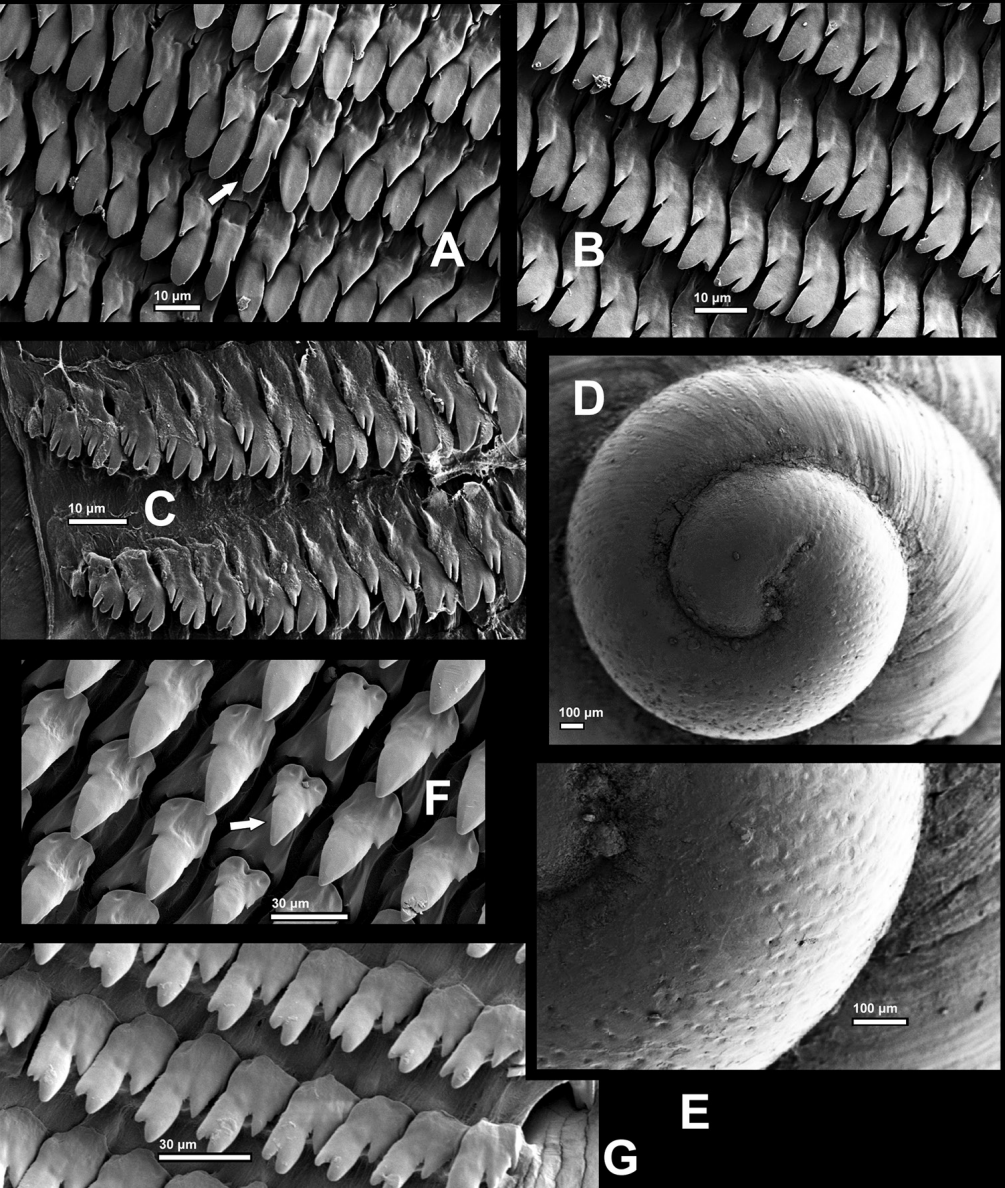
SEM images **A–E***Sinorachis baihu* Wu & Chen, gen. and sp. nov. HBUMM08296-specimen 2, paratype: **A** radula, showing central tooth (arrowed) and several lateral teeth **B** radula, showing tricuspid lateral teeth **C** radula, showing most lateral part **D** embryonic shell **E** magnified embryonic shell, showing some pits **F–G** radula of *Laeocathaica prionotropis* Möllendorff, 1899, HBUMM08299-spec.1: **F** showing central tooth (arrowed) and nearby lateral teeth **G** marginal part of radula.

***Radula*** (Fig. 6A–C): Teeth arranged in transversal rows, each row containing about 151 (75-1-75) closely arranged teeth. Central tooth tricuspid, narrowly tongue-shaped (Fig. 6A). Lateral teeth slightly thickened at inner edge; bicuspid (L1–2 or L1–3) (Fig. 6A), tricuspid (from L2 or L3 on) (Fig. 6A, B) and gradually transformed to marginals with one endocone and three or four ectocones (Fig. 6C).

**Figure 7.**
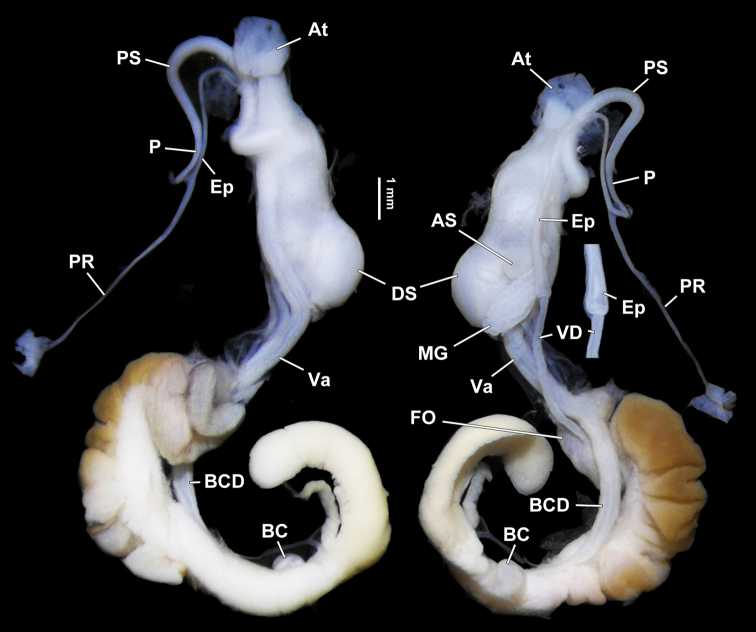
*Sinorachis baihu* Wu & Chen, gen. and sp. nov., HBUMM08296-specimen 1, holotype. Both sides of genitalia. The portion with the demarcation between epiphallus and vas deferens is magnified.

**Figure 8.**
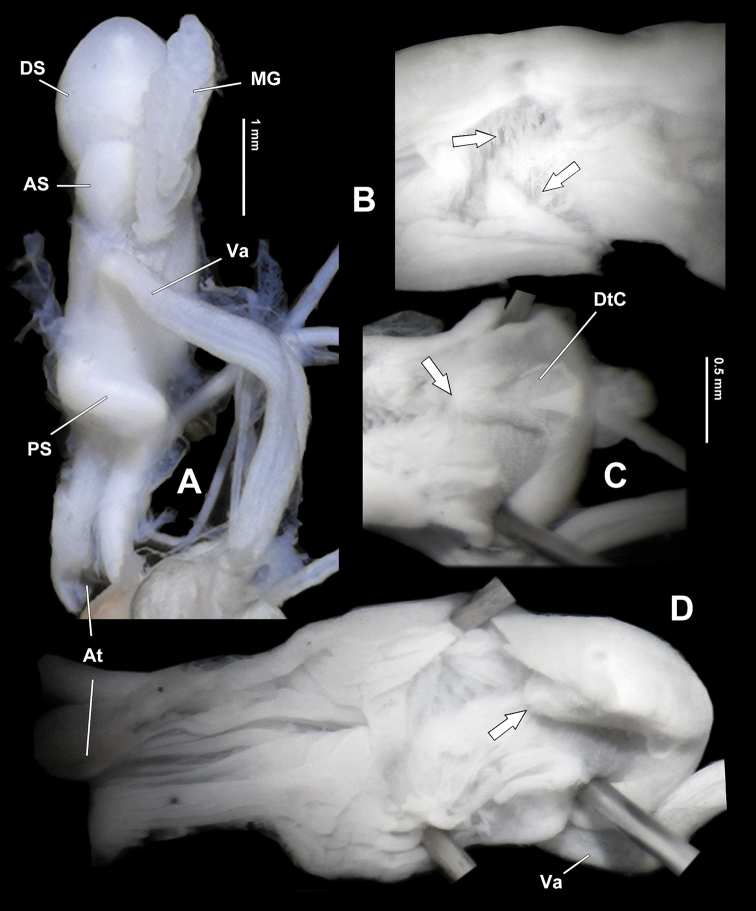
*Sinorachis baihu* Wu & Chen, gen. and sp. nov., HBUMM08296-specimen 1, holotype **A** bottom view of dart sac apparatus **B** partial dorsally exposed dart sac apparatus, showing arrowed poly-layered structure **C** distal part of dorsally exposed dart sac apparatus, showing opened love dart chamber, with opening of the love dart chamber arrowed **D** dorsally exposed dart sac apparatus with opening of love dart chamber arrowed.

***Genital system*** (Figs 7, 8). Penis sheath long, covering approximately 3/4 of penis. Penis thin; externally simple; internally with three pilasters. Epiphallus subequal to penis in length; without epiphallic papilla. Flagellum absent. Vas deferens ca. 1/2 length of epiphallus; of even thickness. Epiphallus and vas deferens sharply demarcated (Fig. 7). Dart sac apparatus large in size; distal 1/3 with a distinct accessory sac ventrally that is internally solid. Love dart very short, approximate 0.7 mm long; sharply tapering from distal end; transparent. Mucous gland with one common peduncle; simply branched. Vagina as long as penis. Bursa copulatrix small, ball-shaped.

###### Measurements of holotype.

DS–5.9 mm long, 1.5 mm broad; DtC–0.7 mm; MG–2.7 mm; P–7.3 mm; Ep–7.5 mm; VD–3.8 mm; PR–5.5 mm; Va–5.7 mm; FO–3.1 mm; BC plus BCD–5.7 mm.

###### Etymology.

This species is named after *baihu* (=白虎in Chinese, means white tiger) which is the totem of the local Tujia people.

###### Type locality.

Lichuan, only known from the type locality (Fig. 1).

###### Distribution.

Hubei.

###### Ecology.

**Figure 9.**
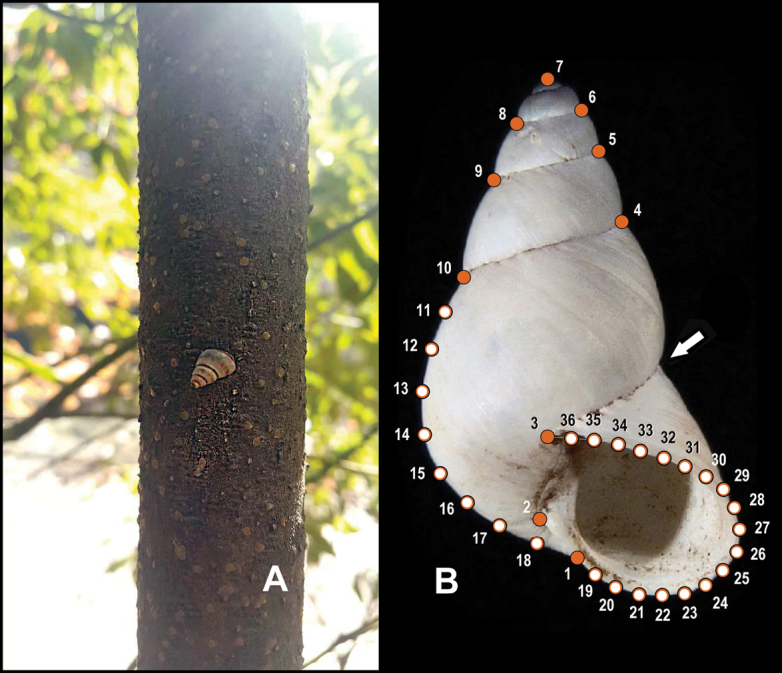
**A** habitat of *Sinorachis baihu* Wu & Chen, gen. and sp. nov., HBUMM08296-specimen 2, paratype **B** a diagram showing design of landmarks (solid orange dots) and semi-landmarks (empty orange dots). Arrow indicates the crossing point made by the right profile and the last part of the suture.

This species was only found on the trunk of a tree (Fig. 9A).

###### Taxonomic remarks.

The new species and the two species that were once placed in the genus *Rachis* share many conchological features. However, typical sigmurethrous pallial complex (Solem 1985) is observed in the new species. The new species is slightly smaller than and obviously thinner than *Sinorachis onychinus* (height 16 mm, diam. maj. 11 mm: Heude 1885: 114, pl. 30, fig. 5). The new species can be distinguished from *S. onychinus* by having evenly distributed pits each centrally with a hump on the embryonic shell. In *S. onychinus*, the embryonic shell is smooth on the first 0.5 whorl, and is axially wrinkled on the subsequent protoconch whorls (0.5–1.25 whorl). On the remaining embryonic whorls, the sculpture is shown as evenly distributed tiny pits [examined material: SMF42825, SMF42826: Patung, Hupei, Mlldff. G., Slg. Kobelt u. Bttgr. SMF42827: *Sinorachis onychinus* (not paratypes of *Rhachis chalcedonicus* as mentioned in Yen 1939: 91, pl. 8, fig. 47), SW Hubei, Gredler G., Slg. Mlldff. SMF1045593] that become weak or disappear altogether.

In pulmonates the presence of a jaw is a ubiquitous characteristic related to herbivorous/ detritivorous/ fungivorous foraging strategies, while the absence of a jaw is correlated with predation/carnivorous foraging strategies (Mordan and Wade 2008). In addition, the absence of a jaw also occurs in some non-carnivorous groups, such as Achatinellidae, which are fungivorous and have the jaw weakly developed or absent (Schileyko 1998a). The comparison between the new species and *Laeocathaica prionotropis* Möllendorff, 1899 (Bradybaeninae) (Fig. 6F, G. HBUMM08299-spec. 1, Bikou, Wenxian, Gansu. Coll. Li, Q., April 2019) indicates they are two different types of radula. The latter species, a typical ground-dweller, is herbivorous snail, which has the robust cone-shaped and sparsely arranged radular teeth that seem to be typical in bradybaenine snails (e.g., compare it with fig. 4 in Páll-Gergely and Hunyadi 2016), while the new species has more slender and densely arranged radular teeth, which suggest the diet range of this species might not cover large plants and animals.

**Figure 10.**
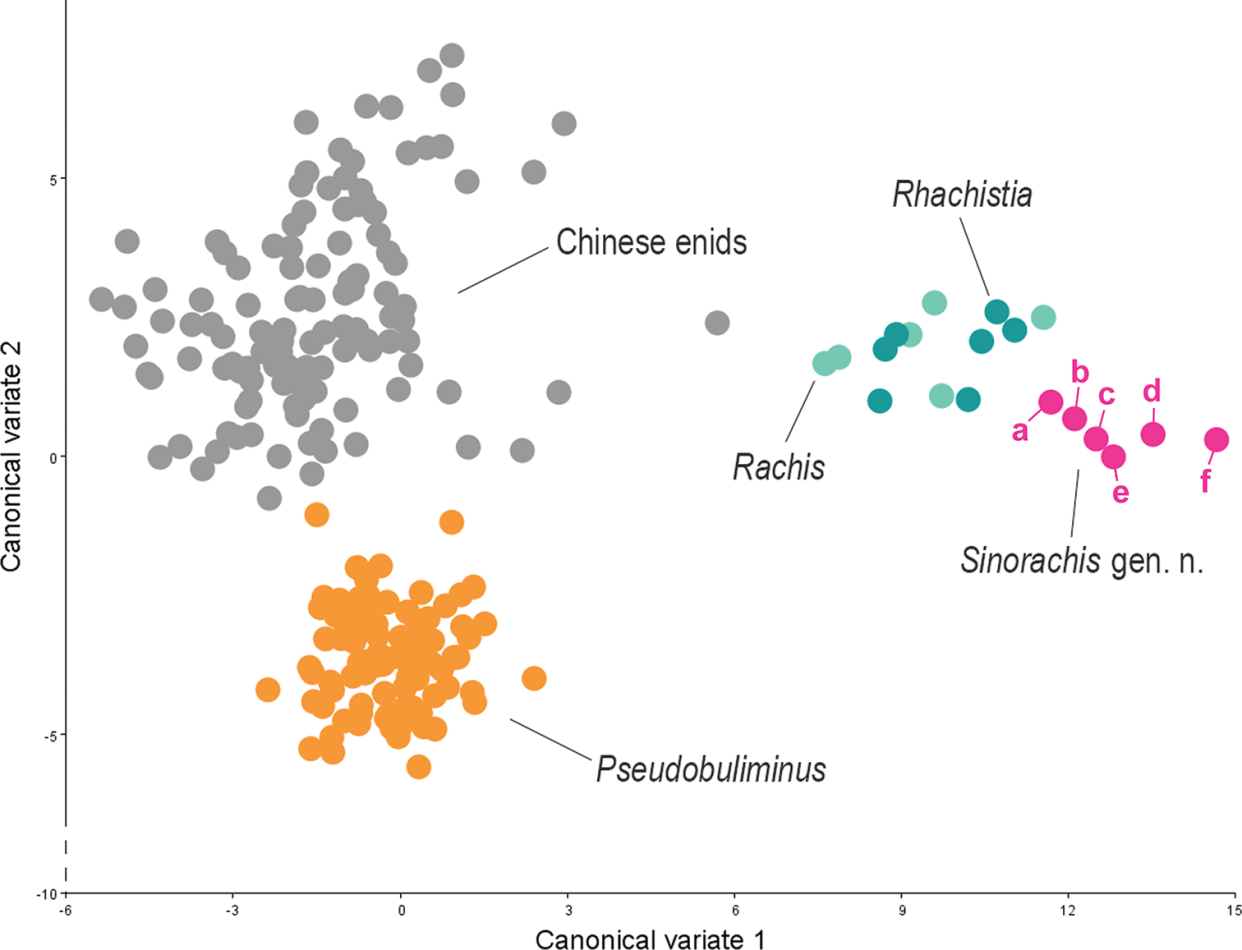
Scatter plot of canonical variate 1 against canonical variate 2 (Canonical Variate Analysis), showing the shell morphological relationship among *Rachis* Albers, 1850 (bright blue dots), *Rhachistia* Connolly, 1925 (grey-blue dots), Chinese *Pseudobuliminus* spp. (orange dots), Chinese enid species (grey dots) and *Sinorachis* gen. nov. (pink dots). a, *Sinorachis onychinus* (Heude), SMF42826; b, *S. onychinus*, SMF104593; c, *S. baihu* Wu & Chen, gen. and sp. nov., holotype; d, *S. onychinus*, SMF42825; e, *S. aureus* (Heude), based on an image from Heude (1890); f, *S. onychinus*, SMF42827.

## Supplementary Material

XML Treatment for Sinorachis

XML Treatment for Sinorachis baihu

## Appendix 1

Brief information of specimens and photographs used in the geometric morphometric analysis.

***Pseudobuliminus* (s. l.) Gredler, 1886 + *Stenogyropsis* Möllendorff, 1899**

*Pseudobuliminus achatininus* (Möllendorff, 1899)

SMF9026 (1, number of specimen), SMF9027 (2), HBUMM00523 (2): China (label lost), HBUMM04429 (2): (Gansu), HBUMM05421 (1): Gansu.

*Pseudobuliminus buliminoides* (Heude, 1882)

SMF24668 (2).

*Pseudobuliminus buliminus strigatus* (Möllendorff, 1899)

SMF9011 (1), HBUMM01187 (2): Sichuan, HBUMM04458 (2): Gansu, HBUMM04613 (2): Gansu, HBUMM05506 (1): Gansu, HBUMM05558 (1): Gansu, HBUMM05562 (2): Gansu, HBUMM05773 (1): Gansu.

*Pseudobuliminus cerasinus* (Gredler, 1892)

SMF95044 (2).

*Pseudobuliminus certus* (Zilch, 1938)

SMF24673 (1), SMF24751 (4).

*Pseudobuliminus chineensis* (Bavay & Dautzenberg, 1908)

SMF186520 (2).

*Pseudobuliminus cristatellus* (Möllendorff, 1902)

HBUMM06746B (2): Sichuan, HBUMM00503 (2): Gansu.

*Pseudobuliminus gracilispirus* (Möllendorff, 1899)

SMF9028 (1), SMF9029 (2), HUMM04456 (2): Gansu.

*Pseudobuliminus hirsutus* (Möllendorff, 1899)

SMF9022 (1), SMF9023 (3), HBUMM05535 (1): Gansu, HBUMM05582 (1): Gansu, HBUMM06565 (2): Sichuan, HBUMM06677B (1): Gansu.

*Pseudobuliminus incertus* (Schmacker & Böttger, 1891)

SMF24675 (1), SMF36015 (1), SMF36951 (1), SMF96649 (1).

*Pseudobuliminus meiacoshimensis* (Adams & Reeve, 1850)

SMF294303 (2).

*Pseudobuliminus paradoliolus* Zilch, 1951

SMF36081 (2), SMF50090 (1).

*Pseudobuliminus piligerus* (Möllendorff, 1899)

SMF9024 (1), SMF9025 (1), HBUMM04428 (2): Gansu, HBUMM05428 (2): Gansu, HBUMM05556 (2): Gansu, HBUMM06745B (1): Gansu, HBUMM06902B (2): Sichuan, HBUMM04432 (2): Gansu, HBUMM04448 (2): Gansu.

*Pseudobuliminus subcylindricus* (Möllendorff, 1899)

SMF9018 (3), SMF9019 (1), SMF9021 (1), HBUMM04449 (2): Gansu.

*Pseudobuliminus subdoliolus* (Haas, 1935)

SMF42549 (1), SMF9323 (1).

*Pseudobuliminus superbus* (Möllendorff, 1888)

SMF9147 (1),

*Pseudobuliminus turritus* (Gude, 1900)

SMF96652 (3), SMF24682 (2)

*Stenogyropsis potanini* (Möllendorff, 1899)

SMF9032, SMF9034 (2), HBUMM05404 (1): Gansu, HBUMM05596 (2): Gansu, HBUMM05643 (2): Gansu, HBUMM05698 (2): Gansu, HBUMM05701 (2): Gansu, HBUMM05722 (2): Gansu

***Rachis* Albers, 1850**

*Rachis zonulata* (L. Pfeiffer, 1846)

SMF74313 (3).

*Rachis* sp.

SMF426914 (2): original label “*Rachis succincta*”.

*Rachis punctatus* (Anton, 1838)

After fig. 39A (Raheem et al. 2014).

***Rhachistia* Connolly, 1925**

*Rhachistia bengalensis* (Lamarck, 1822)

After fig. 39B (Raheem et al. 2014).

*Rhachistia praetermissus* (W.T. & H.F. Blanford, 1861)

After fig. 39C–E (Raheem et al. 2014).

*Rhachistia pulcher* (Gray, 1825)

After figs 39F, 40A (Raheem et al. 2014).

*Rhachistia trutta* (Blanford, 1866)

After fig. 40B (Raheem et al. 2014).

**Chinese enid species**

After Wu (2018) except *Funiculus songi* Wu & Xu, 2011.
